# Large scale analysis of the SARS-CoV-2 main protease reveals marginal presence of nirmatrelvir-resistant SARS-CoV-2 Omicron mutants in Ontario, Canada, December 2021–September 2023

**DOI:** 10.14745/ccdr.v50i10a05

**Published:** 2024-10-03

**Authors:** Venkata Duvvuri, Fatima Shire, Sandra Isabel, Thomas Braukmann, Shawn Clark, Alex Marchand-Austin, Alireza Eshaghi, Hina Bandukwala, Nobish Varghese, Ye Li, Karthikeyan Sivaraman, Hadia Hussain, Kirby Cronin, Ashleigh Sullivan, Aimin Li, Austin Zygmunt, Karam Ramotar, Julianne Kus, Maan Hasso, Antoine Corbeil, Jonathan Gubbay, Samir Patel

**Affiliations:** 1 Public Health Ontario, Toronto, ON; 2 Department of Laboratory Medicine and Pathobiology, Temerty Faculty of Medicine, University of Toronto, Toronto, ON; 3 Division of Biostatistics, Dalla Lana School of Public Health, University of Toronto, Toronto, ON; 4 Department of Family Medicine, University of Ottawa, Ottawa, ON

**Keywords:** SARS-CoV-2, Omicron, Paxlovid, nirmatrelvir-ritonavir, main protease gene (M^pro^), *in vitro* resistant mutations, genomic surveillance, Ontario

## Abstract

**Background:**

In response to the COVID-19 pandemic, a new oral antiviral called nirmatrelvir-ritonavir (Paxlovid^TM^) was authorized for use in Canada in January 2022. *In vitro* studies have reported mutations in M^pro^ protein that may be associated with the development of nirmatrelvir resistance.

**Objectives:**

To survey the prevalence, relevance and temporal patterns of M^pro^ mutations among SARS-CoV-2 Omicron lineages in Ontario, Canada.

**Methods:**

A total of 93,082 M^pro^ gene sequences from December 2021 to September 2023 were analyzed. Reported *in vitro* M^pro^ mutations were screened against our database using in-house data science pipelines to determine the nirmatrelvir resistance. Negative binomial regression was conducted to analyze the temporal trends in M^pro^ mutation counts over the study time period.

**Results:**

A declining trend was observed in non-synonymous mutations of M^pro^ sequences, showing a 7.9% reduction (95% CI: 6.5%–‬9.4%; *p*<0.001) every 30 days. The P132H was the most prevalent mutation (higher than 95%) in all Omicron lineages. *In vitro* nirmatrelvir-resistant mutations were found in 3.12% (n=29/929) Omicron lineages with very low counts, ranging from one to 19. Only two mutations, A7T (n=19) and M82I (n=9), showed temporal presence among the BA.1.1 in 2022 and the BQ.1.2.3 in 2022, respectively.

**Conclusion:**

The observations suggest that, as of September 2023, no significant or widespread resistance to nirmatrelvir has developed among SARS-CoV-2 Omicron variants in Ontario. This study highlights the importance of creating automated monitoring systems to track the emergence of nirmatrelvir-resistant mutations within the SARS-CoV-2 virus, utilizing genomic data generated in real-time.

## Introduction

Nirmatrelvir-ritonavir (brand name Paxlovid^TM^, Pfizer Inc.) is an orally administered antiviral therapy. This combination received an Emergency Use Authorization from United States Food and Drug Administration in December 2021 (1–3). Nirmatrelvir-ritonavir was subsequently approved by Health Canada for adults with COVID-19 who were at high risk of progressing to severe disease in January 2022 (4,5). Nirmatrelvir (PF-07322332), an active component of Paxlovid, is a novel inhibitor of the SARS-CoV-2 3-chymotrypsin-like protease (3CL^pro^) or main protease (M^pro^, also known as non-structural protein, nsp5), which is critical for viral replication and assembly. This inhibitory mechanism prevents the production of new viruses in infected cells (6). Importantly, nirmatrelvir is highly specific to the viral protease, which reduces the risk of off-target effects on human proteases (7). Ritonavir inhibits the cytochrome P4503A4 (CYP3A4) enzyme, a major human hepatic drug-metabolizing enzyme, increasing the plasma concentrations of nirmatrelvir *in vivo* (8).

Clinical efficacy studies on nirmatrelvir-ritonavir reported fewer visits to the emergency department, lower hospitalizations, and lower all-cause mortality in patients infected with SARS-CoV-2 variants of concern (Delta B.1.617.2 and Omicron B.1.1.529, BA.2, BA2.12.1, BA.4 and BA.5) (9–12). A retrospective observational study from Ontario, Canada, reported a significant reduction in hospital admission from COVID-19 and all-cause mortality among outpatients who used nirmatrelvir-ritonavir between April and August 2022, with greater benefits being noted among individuals who were under-vaccinated or unvaccinated and 70 years of age and older (13). The Canadian Nosocomial Infection Surveillance Program found that 13% (n=490/3,731) of adult patients with COVID-19 received nirmatrelvir-ritonavir, either at admission or during hospitalization in Canada, although the results on treatment efficacy remain unreported (14).

The therapeutic effectiveness of nirmatrelvir-ritonavir can be influenced by the emergence of resistant variants. Given the continuous evolution of the SARS-CoV-2 virus and selection pressures from the introduction of nirmatrelvir-ritonavir, resistance is likely to emerge (15). Evidence of *in vitro* nirmatrelvir-resistant SARS-CoV-2 variants (16–18), variable potencies of nirmatrelvir to different human coronaviruses (19) and resistance of other viruses to protease inhibitors (20) support the need for continuous monitoring of SARS-CoV-2 M^pro^ gene sequences to quickly identify mutations that may affect nirmatrelvir’s potency. Such genomic surveillance could provide insights into the mechanisms of antiviral evasion that are crucial for policy guidelines and in the development of next-generation M^pro^ inhibitors (18).

The purpose of this study was to survey the prevalence, relevance and temporal patterns of M^pro^ mutations among circulating SARS-CoV-2 lineages in Ontario. First, we conducted a scientific and grey literature review (May 2022 to August 2023) to compile a list of M^pro^ mutations that have been characterized as conferring *in vitro* resistance to nirmatrelvir (21). This complied list was subsequently used to identify the presence of nirmatrelvir-resistant mutations within the dataset. We then analyzed 93,082 M^pro^ sequences derived from SARS-CoV-2 Omicron-positive clinical specimens sequenced in Ontario between December 2021 and September 2023.

## Methods

### Clinical specimen selection and SARS-CoV-2 whole genome sequencing

Diagnostic laboratories in Ontario provided a proportion of all SARS-CoV-2 positive clinical specimens to designated whole-genome sequencing (WGS) laboratories as part of the Ontario COVID-19 Genomics Network (22). The acceptable criteria for WGS sampling included a SARS-CoV-2 polymerase chain reaction (PCR) cycle threshold (Ct) of 30 or fewer and a sufficient sample volume. The sampling proportion ranged from 10% to 100% and was adjusted over time based on projected case counts and Ontario COVID-19 Genomics Network sequencing capacity from December 2021 to September 2023. The diagnostic PCR testing for SARS-CoV-2/COVID-19 was restricted to high-risk populations (23,24) and, as such, representative surveillance pertains only to those populations tested at the time of sampling.

### SARS-CoV-2 main protease sequences

Raw sequence data from the Illumina platform were analyzed using ARTIC pipeline v1.7 (the Ontario Institute for Cancer Research pipelines) and ARTIC primer scheme version 4.1. Post-analysis quality filtering was performed using ncov-tools version 1.8. Samples were annotated for lineage with Pangolin v4.3 using constellations v.0.1.12 (Pangolin-assignment v1.15.1, Scorpio 0.3.17, and usher 0.5.6). The ARTIC nanopolish v1.3.0-dev (+0.3.1 patch) pipeline and associated ncov-tools version were used for samples sequenced on the nanopore platform. All available M^pro^ gene sequences of SARS-CoV-2 Omicron (n=93,082 unique sequences) were collected between December 1, 2021, and September 21, 2023, from Public Health Ontario’s SARS-CoV-2 WGS database (PHO-SARS-CoV-2 WGS database). These M^pro^ sequences were screened against the reference SARS-COV-2 genome, Wuhan-Hu-1 (accession no. NC_045512.2), to identify both synonymous and non-synonymous mutations across all Omicron lineages.

### Temporal tracking of main protease mutations in Omicron lineages

An in-house data science pipeline was developed in Python v.3.9.16 to track the temporality and prevalence of observed M^pro^ mutations among Omicron lineages in Ontario. A generalized additive model with restricted cubic spline was fit on the log transformed mutation count. We examined the patterns of the M^pro^ non-synonymous mutations over the study time-period; based on these patterns, a negative binomial regression (R package mgcv v.1.9-0) was used to model the decline of the number of non-synonymous mutations over time.

## Results

A total of 93,082 M^pro^ gene sequences corresponding to 929 Omicron lineages of SARS-CoV-2 from Ontario were analyzed. Omicron lineages were grouped by their prevalence of total sequences analyzed as low (less than one percent) or high (greater than or equal to one percent). Twelve SARS-CoV-2 lineages were categorized with high prevalence. The five lineages with the highest prevalence during defined period were: BA.1.1 (9.3%), XBB.1.5 (8.3%), BQ.1.1 (7.8%), BA.2 (7.4%) and BA.5.2.1 (6.0%).

**Figure 1:**
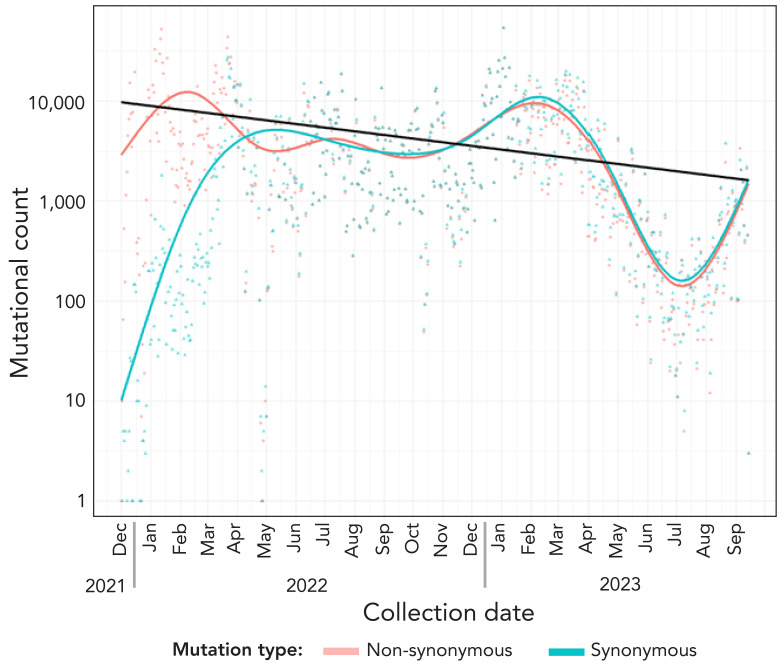
Temporal trends of non-synonymous and synonymous mutations using restricted cubic spline across mutations observed in the main protease (M^pro^) nucleotide sequences of SARS-CoV-2 Omicron lineages circulated in Ontario, Canada, December 2021–September 2023^a^ Abbreviation: M^pro^, main protease ^a^ Each solid circle or dot represents one mutation and is colour-coded based on the M^pro^ protein structural details ((25)). The mutational count is the observed absolute value of mutations at each position. Log transformed Y-axis presents mutational counts

**Figure 2:**
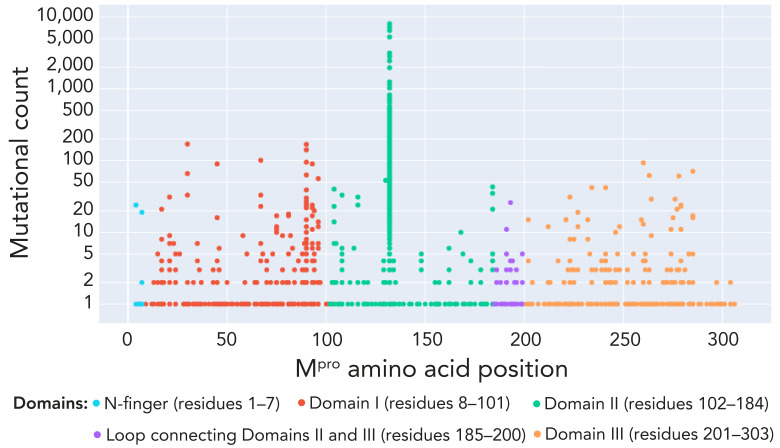
Burden of non-synonymous mutations observed in the main protease (M^pro^) of SARS-CoV-2 Omicron lineages circulated in Ontario, Canada, December 2021–September 2023^a^ Abbreviation: M^pro^, main protease ^a^ Each solid circle or dot represents one mutation and is colour-coded based on the M^pro^ protein structural details ((25)). The mutational count is the observed absolute value of mutations at each position. Log transformed Y-axis presents mutational counts

We studied the evolution of M^pro^ nucleotide sequences of SARS-CoV-2; we observed cyclic variations for total counts for both synonymous (no change in protein sequence) and non-synonymous (change in protein sequence) mutations. The negative binomial regression on M^pro^ non-synonymous mutations showed a 7.9% (95% CI: 6.5%–9.4%; *p*<0.001) decrease in mutation counts every 30 days (**Figure 1**). The non-synonymous mutational burden, with sequences carrying at least one mutation across the M^pro^ protein sequence, accounted for approximately 67.7% (207 AAs/306 AAs of M^pro^) (**Figure 2**). **Table 1** presents details of low and high prevalent lineages with M^pro^ non-synonymous mutations reported in at least 10 sequences of the total sequence data collected for each lineage. For example, the T21I mutation is observed in 31 of 2,801 total BA.2.12.1 sequences during the study period. Only six mutations, L67, L75, K90, A116, P184 and R279, were found to be common in both high and low-prevalent lineages; however, none of these mutations were relevant to the reported *in vitro* nirmatrelvir-resistant mutations.

**Table 1:** M^pro^ non-synonymous mutations with at least 10 observed in the SARS-CoV-2 Omicron lineage sequences, Ontario, Canada, December 2021–September 2023

M^pro^ structure region	Mutation^a^	Pango lineage	Lineage prevalence^b^	Total sequences	Count of sequences with mutation	Frequency^c^	Observation from prior literature (reference)
N-finger (1 to 7 AA)	R4K	BQ.1.2	Low	267	24	8.99	Mutation contributes to M^pro^ dimerization (26)
	A7T	BA.1.1	High	8,143	19	0.23	Mutation contributes to M^pro^ dimerization (27–30))
Domain I (8 to 101 AA)	M17V	XBB.1.5	High	7,281	18	0.25	-
	T21I	BA.2.12.1	High	2,801	31	1.11	*In vitro* study reported as founder or precursor mutation (18)
	L30I	BQ.1.3	Low	33	33	100	L30F, an *in vitro*-reported nirmatrelvir-resistant mutation (9) but L30I has not been tested
		BQ.1.3.1	Low	170	170	100	
		BQ.1.3.2	Low	66	66	100	
	T45N	BE.4	Low	90	90	100	-
		BE.4.1	Low	16	16	100	
		CQ.2	Low	16	16	100	
	L67S	BA.5.2.1	High	5,312	23	0.43	-
	L67V	BF.14	Low	46	32	69.57	-
		BQ.1.1.40	Low	448	101	22.54	
	L75F	BA.1.1	High	8,143	11	0.14	-
		BA.2	High	6,551	12	0.18	
		BA.4.6	High	1,250	17	1.36	
		BA.5.5	Low	705	10	1.42	
	S81C	XBB.1.5	High	7,281	18	0.25	-
	K90R	BA.1.1	High	8,143	95	1.17	Prevalent mutation in Beta (B.1.351) variants ((27))
		BA.2	High	6,551	140	2.14	
		BA.2.12.1	High	2,801	23	0.82	
		BA.5.2	High	3,083	24	0.78	
		BA.5.2.1	High	5,312	25	0.47	
		BQ.1	High	1,987	12	0.6	
		BQ.1.1	High	6,513	39	0.6	
		XBB.1.5	High	7,281	27	0.37	
		BA.2.3	Low	751	166	22.1	
		BA.5.9	Low	70	10	14.29	
		BF.14	Low	46	29	63.04	
		BF.21	Low	104	12	11.54	
		BQ.1.1.51	Low	133	15	11.28	
	T93I	BA.2.12.1	High	2,801	11	0.39	-
		XBB.1.5	High	7,281	6	0.08	
	A94V	BU.1	Low	20	20	100	-
	P96S	BQ.1.1	High	6,513	56	0.86	-
	P96L	XBB.1.5	High	7,281	14	0.19	-
Domain II (102 to 184 AA)	V104I	BN.1.4	Low	15	14	93.33	-
	P108T	BA.5.2.1	High	5,312	33	0.62	-
	A116V	XBB.1.5	High	7,281	31	0.43	-
	A116T	BQ.1.2	Low	267	24	8.99	-
	M130L	BA.5.2.9	Low	534	53	9.93	-
	P168S	BA.5.1	High	2,452	10	0.41	Prevalent mutation in pre-Omicron lineages (17)
	P184S	BA.1.1	High	8,143	21	0.26	-
		BA.2.3	Low	751	43	5.73	-
	P184L	BQ.1.14	Low	250	35	14	-
Loop (185 to 200 AA)	A193V	XBB.1.16.1	Low	227	4	1.76	-
Domain III (201 to 303 AA)	V202I	BQ.1.2.3	Low	289	15	5.19	-
	V212I	FL.7	Low	17	12	70.59	-
	N221S	BQ.1.22	Low	112	15	13.39	-
	F223L	BN.1.3.1	Low	31	31	100	-
	L227F	BA.5.2.1	High	5,312	19	0.36	-
		BF.7	High	999	12	1.2	
	L232F	BA.5.2	High	3,083	14	0.45	-
	A234V	BQ.1.13	Low	435	42	9.66	-
	P241L	XBB.1.5	High	7,281	42	0.58	-
	H246Y	BA.5.1.15	Low	10	10	100	-
	D248N	BU.1	Low	20	12	60	-
	A260V	BQ.1.1	High	6,513	92	1.41	No impact shown on the reducing drug potency in biochemical assay (2,3)
	D263A	BQ.1.1	High	6,513	62	0.95	-
	M264I	XBB.1.5	High	7,281	29	0.4	-
	N274T	BQ.1.1	High	6,513	11	0.17	-
	N274S	BQ.1.14	Low	250	10	4	-
	G275S	BN.1.5.2	Low	18	16	88.89	-
	M276I	BA.5.1.2	Low	154	28	18.18	-
	N277I	BA.5.1.23	Low	236	21	8.9	-
	G278R	BF.7	High	999	58	5.81	-
	R279C	BF.7	High	999	23	2.3	-
		BA.5.5	Low	705	22	3.12	-
		BF.1	Low	95	11	11.58	-
	A285T	BA.2	High	6,551	16	0.24	Mutation contributes to M^pro^ dimerization ((26)) and potential decrease in M^pro^ catalytic efficiency (31)
		BF.1	Low	95	11	11.58	-

Abbreviations: M^pro^, main protease; -, not applicable

^a^ P132H, given its predominance in Omicron lineages, has been excluded from this table

^b^ Lineage prevalence: Omicron lineages were grouped by their prevalence of total sequences analyzed as low (less than one percent) or high (greater than or equal to one percent)

^c^ Formula used to calculate the frequency is: (count of sequences with mutation/total sequences)*100

### Pattern of documented highly prevalent mutations in SARS-CoV-2 Omicron lineages, Ontario

Of the nine most prevalent M^pro^ mutations in SARS-CoV-2 (G15S, T21I, K88R, L89F, K90R, P108S, P132H, L205V and A260V) (17,26,27,32)), albeit with unaltered susceptibility to nirmatrelvir (2,3), only P132H accumulated at a noticeable frequency, eventually accounting for more than 95% in the Omicron lineages in Ontario ((27)). The K90R mutation was observed in the following Omicron lineages: BA.1.1, BA.2, BA.2.12.1, BA.5.2, BA.5.2.1, BQ.1, BQ.1.1 and XBB.1.15 (within lineage rates ranged from 0.37% to 2.14%). While the A260V substitution was observed in 1.41% (n=92/6,513 sequences) of BQ.1.1 variants circulated in 2022, the T21I mutation accumulated in BA.2.12.1 lineage with 1.1% (n=31/2,801 sequences) mutational frequency.

### Low prevalence and no temporality of nirmatrelvir drug resistance in SARS-CoV-2 Omicron lineages, Ontario

**Figure 3:**
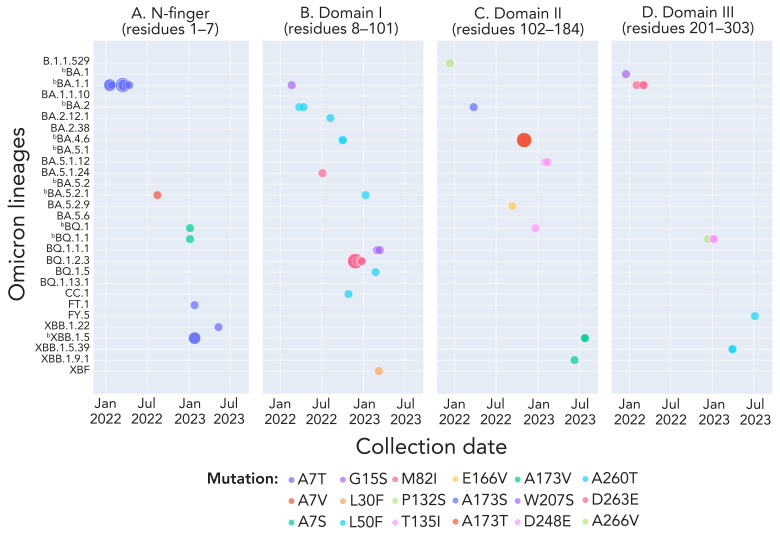
*In vitro* characterized nirmatrelvir-drug resistant mutation accumulation in SARS-CoV-2 Omicron lineages and its temporal patterns in Ontario, Canada, December 2021–September 2023^a,b^ ^a^ Each solid circle or dot represents a count of the corresponding colour-coded M^pro^ mutation and the size of solid circle denotes its count value. The listed M^pro^ mutations correspond to the M^pro^ structural regions of the mutation ((25)) ^b^ Denotes highly prevalent lineages. Appendix, Table A1 provides counts of each mutation and associated lineages in time

Sixteen of 34 *in vitro* characterized nirmatrelvir-resistant mutations (2,3,32,33)), corresponding to A7T/S/V (M^pro^ N-finger), G15S, L30F, L50F, M82I (M^pro^ Domain I), P132S, T135I, E166V, A173S/T/V (M^pro^ Domain II), Q189K, T196A (loop that connects M^pro^ Domains II and III), W207S, D248E, A260T, D263E and A266V (M^pro^ Domain III), were observed with lineage-specificity (3.12%, n=29/929 lineages) (**Figure 3**). The burden of these mutations ranged from 1 to 19 counts, with A7T being the most frequently observed in BA.1.1 (within lineage rate=0.23%, n=19/8,143 sequences; observed only once in FT.1, XBB.1.22 and XBB.1.5), followed by M82I in nine sequences of BQ.1.2.3. The rest were observed in n=4 sequences of BA.4.6 for the A173T mutation, according to **Appendix, ****Table A1**. Only A7T and M82I exhibited some temporality; A7T was notable during weeks three, four and 10 to 15 in 2022 among the BA.1.1 lineage and M82I during weeks 46 to 51 in late 2022 among the BQ.1.2.3 lineage (Figure 3).

We also examined our database for double (18,21,33–35)), triple (2,3), quadruple and quintuple (2,3) mutants, as have been reported in the literature, since these multiple mutations have the potential to confer synergistic resistance to nirmatrelvir. However, none of these mutations were identified within SARS-CoV-2 lineages circulating at the time of sampling in Ontario.

## Discussion

A comprehensive analysis of SARS-CoV-2 Omicron lineage M^pro^ sequences from Ontario revealed that approximately 3% of lineages (n=29/929) exhibited *in vitro* characterized nirmatrelvir-resistant M^pro^ mutations, without any discernible temporal pattern.

Consistent with the global literature (26,27,32), the missense mutation P132H in the M^pro^ structural Domain II region was the most widespread with higher than 95% prevalence in all Ontario Omicron lineages. In addition, K90R, the most prevalent mutation of Beta variants, was observed with modest prevalence in the Ontario Omicron lineages (27)). However, despite their predominance, these two mutations were not reported to reduce nirmatrelvir potency (2,3). Structural assessments of M^pro^ revealed that both mutations (P132H and K90R) are distal to the nirmatrelvir binding site and, thus, do not alter structural conformation at or around the binding site (34)). The A260V substitution, another highly prevalent M^pro^ mutation observed in BQ.1.1 variants reported as an infrequent natural polymorphism, was flagged in the EPIC-HR clinical trial with impact on nirmatrelvir-resistance pending (2,3).

In our dataset, we observed a low frequency of M^pro^ point mutations, such as T21I, P252L and T304I, which are known to function as “precursor” mutations for the emergence of nirmatrelvir resistance in SARS-CoV-2 (18). These three mutations may independently limit the replication of the SARS-CoV-2 virus ((32)), but no data are available on their potential contribution to resistance. None of the low prevalence mutations observed in our dataset, including A7T and M82I, are implicated in nirmatrelvir-resistance ((35)). Notably, A7 is situated within the N-finger region, known to play a role in dimerization which is crucial to M^pro^ enzyme activity ((28,29)). According to Iketani *et al.* ((30)), variants with mutations of A7 to V/C/S/T have comparable protease activity to wild type. Consistently, structural studies suggest that the alanine substitution by threonine at position 7 only has a modest effect on protease activity of M^pro^, a reduction in efficiency by 1.5 times (29). Altogether, these studies suggest that the A7V/S/T mutations observed in BA.1.1 variants in early 2022 were unlikely to contribute to nirmatrelvir-resistance or protease activity. No known *in vitro* nirmatrelvir-resistant mutations were found (as of September 17 to 30, 2023) in Ontario's recently circulating variants, EG.5.1.1, FL.1.5.1, HV.1, HK.3 and XBB.1.16.6 ((36)).

The declining pattern seen in non-synonymous M^pro^ mutations (Figure 1) suggests the possibility of either a reduced heterogeneity among Ontario’s circulating viral variants or a decreased propensity for the M^pro^ protein to evolve in response to selective pressure (27). Alternatively, Schwartz *et al.* (13) reported only 5% of patients (n=8,876/177,545) had been treated with nirmatrelvir-ritonavir between April 4, 2022, and August 31, 2022, in Ontario. These data, although specific to a brief study period within the timeframe of our study, suggest limited selection pressure, potentially contributing to the lower prevalence of antiviral-resistant Omicron variants observed in the population studied. Overall, our observations suggests that Omicron variants analyzed at the time of study period have not yet developed significant and widespread resistance to nirmatrelvir (37)).

### Strengths and limitations

A major strength of the study is the large scale of the analysis of the M^pro^ sequences from the Omicron lineages that circulated between December 2021 and September 2023 in Ontario. A comprehensive analysis led to insights related to *in vitro* mutations relevant to nirmatrelvir resistance (both mutational frequencies and temporality), protease activity and the identification of mutations of unknown function unique to our dataset that may be investigated further in experimental studies. A key limitation of our study is its generalizability, because only a defined sampling proportion was sequenced at given time (i.e., targeted population for COVID-19 diagnostic testing, proportions of specimens sequenced that vary in time, specimens with PCR Ct of fewer than 30). Because of this stringent criteria for sequencing samples, our study dataset may not be directly representative of M^pro^ sequences of Ontario. Furthermore, a lack of availability of sociodemographic, clinical and treatment data limited the interpretation of our findings in the context of nirmatrelvir-ritonavir treatment.

## Conclusion

Overall, we found very low presence of nirmatrelvir-resistant mutant strains with lack of temporality. Our data suggest that the current use of nirmatrelvir-ritonavir targeting specific populations in Ontario may not provide selective pressure for the emergence of resistant mutants (37)). Finally, this study underpins the need for continuous genomic surveillance and also forms the foundation for the creation of an automated monitoring system designed to track the emergence of nirmatrelvir-resistant mutations within the SARS-CoV-2 virus, utilizing real-time genome data. The ability to track, in near real-time, the frequency of mutations associated with antimicrobial resistance can inform the antimicrobial stewardship necessary to maintain drug efficacy over a longer period.

## Appendix

**Table A1:** Cumulative detection of nirmatrelvir-resistant M^pro^ mutations observed in SARS-CoV-2 Omicron lineages circulated in Ontario, Canada, December 2021–September 2023

M^pro^ mutation	Current evidence	Associated lineage	Year of lineage circulation	*In vitro* reported nirmatrelvir-resistant mutations in each lineage
A7T	Post-treatment emergence	BA.1.1	2022	19
		FT.1	2023	1
		XBB.1.22	2023	1
		XBB.1.5	2023	2
A7V	Post-treatment emergence	BA.5.2.1	2022	1
A7S	Post-treatment emergence	BQ.1	2023	1
		BQ.1.1	2023	1
G15S	Biochemical assay, resistance selection study	BA.1.1	2022	1
		BQ.1.1.1	2023	2
L30F	Post-treatment emergence	XBF	2023	1
L50F	Resistance selection study	BA.2	2022	2
		BA.2.12.1	2022	1
		BA.4.6	2022	2
		CC.1	2022	1
		BA.5.2.1	2023	1
		BQ.1.5	2023	1
M82I	Post-treatment emergence	BA.5.1.24	2022	1
		BQ.1.2.3	2022	9
T135I	Biochemical assay	BQ.1	2022	1
		BA.5.1.12	2023	2
E166V	Biochemical assay, post-treatment emergence	BA.5.2.9	2022	1
A173S	Biochemical assay, cell culture assay	BA.2	2022	1
A173T	Biochemical assay, cell culture assay	BA.4.6	2022	4
A173V	Biochemical assay, cell culture assay	XBB.1.5	2023	2
		XBB.1.9.1	2023	1
Q189K	Biochemical assay	XBB.1.5	2023	1
T196A	Post-treatment emergence	BA.2	2022	1
		BA.2.3	2022	1
		BA.4.1	2022	1
W207S	Post-treatment emergence	BA.1	2021	1
D248E	Biochemical assay	BQ.1.1	2023	3
A260T	Post-treatment emergence	FY.5	2023	1
		XBB.1.5.39	2023	2
D263E	Post-treatment emergence	BA.1.1	2022	3
A266V	Post-treatment emergence	BQ.1.1	2022	1

Abbreviation: M^pro^, main protease
